# Evaluating the technical efficiency of neonatal health service among primary hospitals of northwest Ethiopia: Using two-stage data envelopment analysis and Tobit regression model

**DOI:** 10.1371/journal.pone.0277826

**Published:** 2022-11-17

**Authors:** Anteneh Lamesgen, Amare Miniyihun, Tsegaw Amare

**Affiliations:** 1 Department of Public Health, College of Medicine and Health Sciences, Debre Markos University, Debre Markos, Ethiopia; 2 Department of Health Systems and Policy, Institute of Public Health, College of Medicine and Health Sciences, University of Gondar, Gondar, Ethiopia; Taipei Medical University, TAIWAN

## Abstract

**Introduction:**

Most cases of neonatal mortality are preventable but a substantial number of cases get complicated and become irreversible not only due to scarcity but also due to inefficient utilization of available resources in the health service. However, limited evidence shows the efficiency level of health facilities in Ethiopia. Therefore, this study aimed to assess the technical efficiency of neonatal health service and its associated factors among primary hospitals in three zones of Northwest Ethiopia.

**Methods:**

A facility-based cross-sectional study was conducted among fifteen randomly selected primary hospitals from March 17 to April 17, 2021. Input data on non-salary recurrent costs, salary costs, and the number of beds, and output data on neonatal admissions, outpatient visits, and referrals for the 2019/20 fiscal year were collected using a document review. In the first stage of analysis, input-oriented data envelopment analysis with a variable return-to-scale assumption was employed to estimate the efficiency scores using DEAP 2.1. A Tobit regression model was fitted in the second stage to identify the associated factors with technical efficiency. Variables with a p-value <0.05 were declared as statistically associated factors.

**Results:**

In this study, 80% of the primary hospitals were pure technical efficient whereas 46.67% of the facilities were scale efficient with all of the scale inefficient hospitals operating below their scale. The mean pure technical and scale efficiency score of primary hospitals was 0.948±0.113 and 0.887±0.143, respectively. Total catchment population, incentive packages for the clinical staff, and the educational status of the manager were positively associated with the technical efficiency of hospitals. On the other hand, technical efficiency was negatively associated with the presence of a health facility that provides neonatal health services near the hospital and the distance of the manager’s residence.

**Conclusion:**

Though most of the primary hospitals in Northwest Ethiopia were technical efficient, more than half of them were working below their scale of operation. Our results also indicated that introducing the performance-based provision of incentive packages for clinical staff and employing master’s and above-educated health professionals as a manager might improve the efficient utilization of resources in primary hospitals.

## Background

The neonatal period, the first 28 days of life, is the most crucial period for a child’s well-being. Globally, 2.4 million neonates died with an approximate number of 6,500 deaths every day and a global rate of 17 deaths per 1,000 live births in 2020 [1]. The probability of death in sub-Saharan Africa is ten times higher than in high-income countries [1]. Ethiopia is one of the top ten countries with the highest neonatal mortality worldwide [2]. According to the Ethiopian mini demographic and health survey, neonatal mortality was 33 deaths per 1,000 live births in 2019, [3] which is far from the sustainable development goal (SDG) of 12 deaths per 1,000 live birth to achieve by the end of 2030 [4].

Even though most cases of neonatal death are preventable through preventive and early treatment interventions [5,6], the low performance of health facilities is hindering the acceleration to achieve the SDG in several low-income countries [5]. Evidence showed that a significant number of newborns die at home because of early discharge from the hospital which leads to barriers to access and delays in seeking health care [2]. In addition, neonatal mortality is higher where there are lower performances in maternal and newborn health care [7,8].

The issue of efficiency has become a central issue of policymakers for performance evaluation of the health system in the world. The explosion of interest in measuring health systems’ inputs, activities, and outcomes is attributed to intensified concerns with the costs of health care, increased demands for public accountability, improved capabilities for measuring performances, and less resource allocation for health care [9,10]. For instance, in Ethiopia, only 4.2% share of the gross domestic product was allocated to health in 2016/17. In addition, US$33.2 was allocated per capita health expenditure [11], which is very low compared to the US$86 that is needed to provide essential health services in low-income countries. On the other hand, according to the 2016/17 Ethiopian Health Account study, primary health facilities including district hospitals, health centers and health posts together took the largest (61%) share of total government recurrent expenditure [11].

In this sense, evaluating the efficient utilization of available resources among hospitals has a huge implication for mortality reduction. Technical efficiency is one of the widely used efficiency assessments of health facilities. It is the best-observed practice achieved by the decision-making unit (DMUs) on the conversion of physical inputs into outputs [12]. In Ethiopia, there are indicators of health care inefficiency. For example, the input-based payment system which does not link resource allocation with expected outputs and a gap in the monitoring and evaluation system were major sources of inefficiency [13].

Studies indicated that sex [14], educational level [15,16], experience [15–17], a distance of the residence from the facility of the manager [15,18], patient waiting time [17,19–22], hospitals service years [15,16,19] and presence of clinicians’ on-job training [20,23] were organizational determinants of the technical efficiency. Whereas the hospital’s geographical location [20,24,25], size [14,22,26,27], catchment population [15–17,19,21,28,29], and availability of health facilities within the catchment area [15,16,30] were environmental factors associated with efficient utilization of resources in the health facilities.

Despite the widespread application of technical efficiency [31,32], scarce evidence was generated on the efficient provision of neonatal health services in hospitals. Besides, most studies [17,19,26,33] were not specific and none of the studies was on neonatal health services in primary hospitals though it is crucial for the improvement of neonatal mortality. A study conducted by Yitbarek et al in 2019 [17] revealed that the majority of the health posts and health centres in southwest Ethiopia were providing technically inefficient neonatal health care. But the study failed to include the primary hospitals although the primary hospitals are the primary referral centre and components of the primary health care units in the health systems of Ethiopia. Hence, evaluating the efficient utilization of resources for the most refereed cases like neonatal health cares in hospitals can yield information for evidence-based decision and policy implication such as resource reallocation, payment system audit and so forth [26]. Therefore, this study aimed at evaluating the technical efficiency of neonatal health services among primary hospitals in three zones of Northwest Ethiopia.

## Materials and methods

### Study design and setting

A facility-based cross-sectional study with a quantitative method was conducted from March 17 to April 17, 2021, among primary hospitals in the East Gojjam, West Gojjam, and Awi zones of Northwest Ethiopia. All three zones are located in the Southwest direction of the Amhara region where an estimated population of 22,537,000 inhabitants live in 2021 [34] based on the Ethiopian central statistical agency prediction. Besides, the Ethiopian health care system is structured into a three-tiered health system; the primary, secondary, and tertiary levels of health care. The primary level of health care is composed of primary hospitals, health centres, and health posts. It provides inpatient pediatric care, neonatal intensive care, essential newborn care, community-based newborn care (CBNC), integrated management of neonatal and childhood illness (IMNCI), and integrated community case management (iCCM) of neonatal health services [35]. The three zones contain 18 primary hospitals (eight from East Gojjam, six from West Gojjam, and four from Awi zone)

### Sample size determination and sampling procedure

The World Health Organization (WHO) guideline for assessing the operationality of district health systems [36] was used to determine the sample size. Using a simple random sampling technique three primary hospitals were selected from each zone. To increase the power of estimating the efficiency frontier, six additional hospitals were randomly selected from the remaining primary hospitals to have a total sample of 15 primary hospitals. The number of inputs and outputs included in this study was determined by the calculated number of DMUs (primary hospitals).

## Variables of the study

The dependent variable of this study was the technical efficiency of neonatal health services provision in primary hospitals. Three inputs and three outputs were selected to measure the efficiency of the DMUs which is in line with the rule of thumb suggested that the number of DMUs should be at least twice the sum of inputs and outputs [37,38]. Inputs and outputs used to estimate the technical efficiency were identified after reviewing related studies [16,17,26]. The inputs were human resources (salary of employees), capital (number of beds to offer neonatal health services), and non-salary recurrent expenditures, and the outputs were the number of neonatal outpatient visits, neonatal inpatient admissions, and neonatal referrals. The independent variables of this study were composed of organizational and environmental variables. Organizational variables include service year of the hospital, manager’s educational status, manager’s service year, availability of incentive packages for the clinical staff, and patient waiting time and environmental variables include catchment population, availability of nearby health facilities that offer neonatal health services, and distance of the manager’s residence from the hospital.

### Data collection tools and procedures

Data were collected using a structured and pretested questionnaire. The questionnaire was prepared after reviewing the standards for primary hospitals of the country [39], integrated management of newborn and childhood illness (IMNCI) guideline [40], related studies [16,17,19], and adapting the WHO tool for assessing the operationality of district health systems [36]. It includes items on environmental and organizational-related characteristics of the primary hospitals, inputs for neonatal health services, and neonatal health services outputs. Items on environmental and organizational characteristics were collected using an interviewer-administered questionnaire from the heads of each primary hospital. Input and output data of neonatal health services for the 2012 Ethiopian fiscal year (July 2019 to June 2020) were collected by reviewing documents using checklists. The input and output data were collected reviewing documents from the hospital’s finance office, human resources, planning office, health information technicians and liaison officer.

Since inputs used in health facilities are not directed to specific health services, this study employed a volume-based shared cost estimation method to determine a specific expenditure value for neonatal health services in each primary hospital. This was done by computing a proportion (dividing the total volume of neonatal health service outputs by the overall outputs of the hospital), and all the resources were then multiplied by this proportion to determine the specific costs of neonatal health services. A bottom-up costing approach, which considers the cost of each item and multiplies with the magnitude to get the total cost, from the provider perspective was employed. This method of costing is usually said to be the best costing method [41]. The estimated cost in Ethiopian Birr (ETB) was changed into United States dollar (US$) using the March 2020 exchange rate of one Ethiopian birr to 0.0306 US dollars [20,42].

### Data quality control

Data collectors who have a Bachelor’s degree in health science fields were recruited, and training for two days was given for them before the data collection. The questionnaire was first prepared in English, then translated into Amharic, and then back-translated into English by a language expert to check for its consistency. A pretest of the questionnaire was done at Koladiba primary hospital, which is one of the primary hospitals in the Amhara region but not the study area, and a revision was made to eliminate misunderstandings. During the data collection period, questionnaires were checked every day for their completeness, and feedback was given to the data collectors when they faced challenges.

### Data processing and analysis

After the data collection, data were checked for completeness, cleaned, and entered into Epi-Data version 4.6. The data were exported to Microsoft office excel 2010 to set the total summarized inputs and outputs of each primary hospital. Descriptive statistics were analyzed using STATA version 14, and a two-stage Data Envelopment Analysis (DEA) was performed. In the first stage, the efficiency scores were determined using DEA Program, version 2.1 (DEAP 2.1) developed by Tim Coelli [43]. The arguments among scholars on choosing the model for the second stage of DEA analysis continued [44]. Since the efficiency scores fell within the interval between 0 and 1, the Tobit regression model was considered to be more appropriate for such outcome variable with a censored dataset than the ordinary least square method as it reduces the bias by taking into account explicitly the bounded domain of the DEA efficiency estimates [9,44]. Therefore, we have employed the Tobit regression model against the efficiency scores to identify the significant organizational and environmental factors.

The Tobit coefficients indicate how a one-unit change in an independent variable *X*_*B*_ alters the latent dependent variable y* [44], [45].

y*=β0+Xβ+μ

$$y=\left\{\begin{array}{c} y*\;if\;y*(0,1)\\ 0\;if\;y*\leq 0\\ 1\;if\;y*\geq 1 \end{array}\right\}$$

Where y is the observed variable whereas y* is the latent variable.

The model employed the maximum likelihood given that the assumption of homoscedastic normal disturbances was met. Finally, significant predictors were identified at a p-value of 0.05 level of significance and 95% confidence level.

### Measures of the outcome variable

Frontier efficiency measurement techniques are the commonly used efficiency measures for the productive performances of health care services; these techniques use a production possibility frontier to map a locus of potentially optimal combination of inputs and outputs in the production of a certain DMU, health service organization, which is generally represented by some form of a production function. Frontiers have been estimated using different methods; the two principal techniques are the Stochastic Frontier Analysis (SFA) and DEA, which involve econometric methods and mathematical programming, respectively [43].

SFA is a parametric technique that estimates the efficiency frontier based on the functional relations of inputs and outputs. The main advantage of SFA is considering the statistical noises, such as errors in measurement and the parametric assumptions allow to employ of a statistical test [46]. On the other hand, the common drawback is the pre-specification of the functional form and an explicit distributional assumption for the efficiency term. Besides, this technique uses the price as well as quantity information as the same which may add measurement errors to the estimation results [47].

Whereas DEA is a non-parametric linear programming technique that develops an efficiency frontier based on observed facts to calculate a given organization’s efficiency relative to other organizations’ performance producing the same output. It is a data-oriented approach, for evaluating the performance of a set of DMUs, which convert multiple inputs into multiple outputs. The main advantage of DEA is that it doesn’t need a specification of the functional form of the production function and can accommodate the scale issue. Besides it is easy to implement and utilize as compared to SFA multiple outputs and multiple inputs at the same time. But DEA doesn’t consider the measurement error that leads to classifying all the deviations from the frontier as an indication of inefficiency. In addition, DEA doesn’t offer a statistical framework for modelling the performance of the DMUs [46]. Therefore, in this study, we have used a DEA method attributing to considering multiple outputs and inputs for the production of neonatal health services and intending to measure the scale efficiency of the hospitals.

Technical efficiency in DEA can be defined as the ratio of a weighted sum of outputs of a given DMU divided by a weighted sum of its inputs [9,47–49].


$$Technical\;efficiency=\frac{The\;weighted\;sum\;of\;outputs}{The\;weighted\;sum\;of\;inputs}$$

In the DEA of technical efficiency, if the focus is on reducing inputs of a given health facility input-oriented DEA will be considered. Input orientation assumes that healthcare managers have more control over the inputs than the number of clients arriving for health services. On the other hand, output-oriented DEA of technical efficiency is considered when the emphasis is on expanding outputs from a given level of inputs. It assumes health care managers can attract service users to their facilities through marketing, referrals, or by other means such as reputation on the quality of services [47].

In this study, input-oriented DEA was conducted as the primary hospitals have less power of control to change their outputs than their inputs. In addition, the health managers of the region would prefer to be informed about how much resources were spent inefficiently. This could enable the managers to decide how to tailor resource allocation to their primary hospitals with better-mined efficiency.

Furthermore, there are two types of models designed to measure the technical efficiency of DMUs using DEA. The former, constant return to scale (CRS) (Charnes, Cooper, and Rhodes model) assumes that the scale of operation for a given DMU is not a factor for its productivity, the optimal mix of its inputs and outputs. Later, the CRS model was extended to a more flexible variable returns to scale (VRS) (Banker, Charnes, and Cooper model), which is appropriate when not all DMUs can be considered to operate at an optimal scale, their productivity is dependent on the scale of operation. The value of technical efficiency obtained from a CRS DEA can be decomposed into the scale and pure technical efficiencies; the former is due to the size of the operation whereas the latter is only due to managerial performance [9,43].

The use of the variable return to scale model calculates the efficiency free from bias due to scale efficiency. Hence it is called pure technical efficiency. The VRS model takes two forms, decreasing returns to scale (DRS) and increasing returns to scale (IRS). DRS refer to a DMU that is too large for the volume of activities it conducts. To run at the most productive scale size, a DMU exhibiting DRS has to scale down its scale of operation. In contrast, a DMU with IRS is too small for its scale of operation. If a DMU is exhibiting IRS, it should expand its operation scale to become an efficient DMU [50]. Since financial and regulatory constraints often result in a sub-optimal scale of operations, the VRS assumption of DEA was considered for this study to get a good estimation of efficiency among the primary hospitals.

Using input-oriented DEA with a VRS assumption, primary hospitals’ relative technical efficiency scores in the provision of neonatal health services were obtained by solving the equation depicted elsewhere [17,47,51]. A hospital is said to be technically efficient when the point that represents the optimal mix of its inputs and outputs lies on the frontier line.


$$Efficiency=max\sum_{r}UrYrjo+Uo$$

Subject to

$$\sum_{r}UrYrj-\sum_{r}ViXij+Uo\leq 0;\;j=1\ldots .n$$

$$\sum_{i}ViXijo=1$$

U_r_, V_i_ ≥ 0. Where; Y_rj_ = the amount of output r produced by health facility j, X_ij_ = the amount of input I used by health facility j, U_r_ = the weight given to output r, (r = 1… t and t is the number of outputs), V_i_ = the weight given to input I, (i = 1… m and m is the number of inputs), j_0_ = the health facility under assessment.

### Ethics approval and consent to participate

This study’s proposal was reviewed by the Institutional Review Committee of the institute of public health, the college of medicine and health science, and the University of Gondar and a letter of ethical clearance with a reference number IPH/1435/2013 was received after approval. The title and the ethical clearance were submitted to the Amhara public health institute to receive a letter of support to run the study and facilitate the data collection. The study’s purpose was explained, and written informed consent was obtained from the chief executive officer of each primary hospital.

## Results

### Organizational and environmental characteristics of primary hospitals

In this study, all the fifteen primary hospitals which were providing health services in the 2012 EFY (July 2019 to June 2020) were assessed. Eighty percent of the primary hospitals had started health services before five years. The study showed that all the primary hospitals had more than 100,000 catchment population and 11 (73.33%) hospitals had another health facility that gives neonatal health services near distance (Table 1).

**Table 1:** Organizational and environmental characteristics of primary hospitals towards neonatal health services provision in three zones of Northwest Ethiopia, 2021 (N = 15 DMUs).

Variable	Category	Frequency (N)	Percent (%)
Service year of the hospital	< 5 years	3	20
	≥ 5 years	12	80
Educational status of the head of the hospital	BSc	7	46.67
	≥ Master	8	53.33
Managerial service year of the head of the hospital	< 5 years	11	73.33
	≥ 5 years	4	26.67
Availability of incentive packages for the clinical staff	Yes	7	46.67
	No	8	53.33
Average patient waiting time	< 30 minutes	12	80
	≥ 30 minutes	3	20
Total catchment population of the hospital	≥ 100,000	15	100
Total neonatal catchment population of the hospital	≥ 3370	15	100
Availability of a health facility near the hospital that provides neonatal health services	Yes	11	73.33
	No	4	26.67
Distance between the manager’s residence to the hospital	< 2km	7	46.67
	≥ 2km	8	53.33

### Inputs and outputs

The study showed that the average yearly inputs of non-salary recurrent costs, salary costs, and beds for neonatal health services were US$1426.99, US$4269.57, and 5.8 for the efficient primary hospitals and US$8419.21, US$4624.95, and 9 for the inefficient hospitals, respectively. On average; the efficient hospitals served 301.25 neonatal admissions, 164.83 neonatal outpatient visits, and 68 neonatal referrals in the 2012 EFY (July 2019 to June 2020) (Table 2).

**Table 2:** Inputs for and outputs of neonatal health service provision among efficient and inefficient primary hospitals in three zones of Northwest Ethiopia, 2021 (N = 15 DMUs).

Variables		Efficient			Inefficient		
		Mean	SD	Sum	Mean	SD	Sum
Inputs	Non-salary recurrent cost in US$	1426.99	693.20	17123.89	8419.21	11507.24	25257.62
	Salary cost of staffs in US$	4269.57	2019.27	51234.80	4624.95	1052.55	13874.85
	No of beds	5.8	1.8	70	9	6.92	27
Outputs	Total number of admissions	301.25	161.49	3615	247.53	73.79	742
	Total number of outpatient visits	164.83	102.07	1978	137.33	54.12	412
	Total number of referrals	68	54.52	816	43	8.72	129

The average input of non-salary costs for neonatal health services was 1426.99 for efficient hospitals and 8419.21 for inefficient ones.

### Technical efficiency of neonatal health services provision in primary hospitals

The study revealed that 12 (80%) primary hospitals were pure technical efficiency and seven (46.7%) hospitals were scale efficient.

All the scale inefficient primary hospitals were running below their scale of operations. The mean (±SD) of pure technical efficiency and scale efficiency scores of the primary hospitals was 0.948 (±0.113) and 0.887 (±0.143) respectively (Table 3).

**Table 3:** Efficiency scores of primary hospitals towards the provision of neonatal health services in three zones of Northwest Ethiopia, 2021 (N = 15 DMUs).

DMUs	CRS TE	VRS TE	Scale efficiency	Return to scale
H01	1.000	1.000	1.000	-
H02	1.000	1.000	1.000	-
H03	0.940	1.000	0.940	IRS
H04	1.000	1.000	1.000	-
H05	0.550	0.637	0.864	IRS
H06	1.000	1.000	1.000	-
H07	0.691	0.794	0.871	IRS
H08	1.000	1.000	1.000	-
H09	0.502	0.784	0.640	IRS
H10	0.560	1.000	0.560	IRS
H11	1.000	1.000	1.000	-
H12	1.000	1.000	1.000	-
H13	0.844	1.000	0.844	IRS
H14	0.836	1.000	0.836	IRS
H15	0.744	1.000	0.744	IRS
Mean	0.844	0.948	0.887	
SD	0.189	0.113	0.143	

### The projection of input reduction and output escalations for the inefficient hospitals

The study revealed the expected inputs and outputs needed to be reduced and increased to make the three technically inefficient hospitals (H05, H07, and H09) efficient. Accordingly, the inputs of non-salary recurrent costs, salary costs, and beds for neonatal health services need to be reduced by 86.37%, 28.29%, and 41.48%, respectively. Similarly, outputs of neonatal admissions and neonatal referrals need to be increased by 3.36% and 18.03%, respectively (Table 4).

**Table 4:** The projection of input reduction and output escalation to make the inefficient primary hospitals efficient in three zones of Northwest Ethiopia, 2021 (N = 15 DMUs).

DMUs		Inputs			Outputs	
		Non-salary recurrent cost in US$	Salary cost of staff in US$	No of beds	Total number of admissions	Total number of referrals
H05	Original	1477.66	4601.83	17	279	39
	Projected	940.56	2623.99	7.91	279	62
	Difference	-36.35%	-42.97%	-53.47%	0%	58.97%
H07	Original	2077.86	5688.87	5	300	53
	Projected	1422.18	4514.76	3.97	300	53
	Difference	-31.56%	-20.64%	-20.60%	0%	0%
H09	Original	21702.10	3584.15	5	163	37
	Projected	1079.61	2809.67	3.92	187.95	37.26
	Difference	-95.02%	-21.60%	-21.60%	15.31%	0.70%
Mean	Original	8419.21	4624.95	9	247.33	43
	Projected	1147.45	3316.14	5.27	255.65	50.75
	Difference	-86.37%	-28.29%	-41.48%	3.36%	18.03%

### Factors associated with neonatal health services provision technical efficiency

The study showed that a 100,000 increase in the catchment population of the hospital increases the predicted technical efficiency score by 0.0341 (95% CI: 0.00751, 0.061). The presence of incentive packages for the clinical staff increases the likelihood of technical efficiency of hospitals by 0.19 (95% CI: 0.07, 0.31). The probability of technical efficiency scores of hospitals increases by 0.16 (95% CI: 0.05, 0.26) when the educational status of the manager is master and above.

The presence of a health facility that provides neonatal health services near the hospital decreases the predicted technical efficiency score by 0.21 (95% CI: -0.32, -0.09). The expected technical efficiency score of the hospitals decreases by 0.14 (95% CI: -0.27, -0.008) when the distance from the manager’s residence to the hospital is less than 2km (Table 5). The model was significantly fitted to explain the efficiency of the hospitals (P = 0.004).

**Table 5:** Factors associated with the technical efficiency of neonatal health service among primary hospitals of three zones of Northwest Ethiopia, 2021 (N = 15 DMUs).

Variables	Coefficients	SE	T	P-value	95% CI
Total catchment population	3.41e-^07^*	1.17e-^07^	2.90	0.018	7.51e-^08^, 6.06e-^07^
Service year of the hospital	-0.02	0.01	-1.66	0.132	-0.04, 0.006
Availability of incentive packages (yes)	0.19**	0.05	3.63	0.006	0.07, 0.31
Availability of a health facility that provides neonatal health services near the hospital(yes)	-0.21**	0.05	-4.08	0.003	-0.32, -0.09
Educational status of the manager (≥ master)	0.16**	0.05	3.32	0.009	0.05, 0.26
Distance from the manager’s residence to the hospital (< 2km)	-0.14*	0.06	-2.4	0.040	-0.27, -0.008
Constant	0.98	0.06	15.41	0.000	0.84, 1.13
Sigma	0.06	0.01			0.03, 0.08
Number of observations	15				
LR Chi^2^(6)	18.90				
Pro>chi^2^	0.004				
Log-likelihood	21.32				

*Significant at p-value < 0.05

**significant at p-value < 0.01.

## Discussion

In this study, the average pure technical efficiency score of the primary hospitals was 0.95 (±0.11SD). This shows that if these hospitals run efficiently in their best managerial performance, five percent of their health service inputs would be saved for the constant volume of outputs they produced. The result of this study exhibit similarity with studies conducted in hospitals of northwest Ethiopia [16], Eritrea [22], and Kenya [18], with the mean pure technical efficiency scores of 0.92, 0.97, and 0.91, respectively. However, the finding is higher than studies conducted in Ethiopia [52] among health centres and in Southwest Ethiopia [17] among health posts with a mean pure technical efficiency score of 0.79 and 0.18. Moreover, the result is also higher than study conducted in hospitals in Gambia, where the mean pure technical efficiency score was 0.65 [53]. This might be due to the use of a higher number of DMUs in the earlier study, resulting in lower efficiency scores of health facilities [38] relative to the current study.

This study revealed that the mean scale efficiency score of the primary hospitals was 0.89 (±0.14SD). This shows that if all hospitals were operating on their optimal scale, 11% of the health service inputs would be reduced without changing the number of outputs produced. About 53% of the primary hospitals were scale inefficient, and all the scale inefficient hospitals were running below their scale of operation. This shows that these hospitals had to increase their size of operation to become efficient as their efficient peer hospitals.

Moreover, 12 (80%) of the primary hospitals were found to be technically efficient. This finding is in line with the studies conducted in Eastern Ethiopia [26] and Eritrea [22], where 75% and 68% of public hospitals were technically efficient. However, studies in Southwest Ethiopia [17] and the Tigray region of Ethiopia [54] showed that only 35% and 25% of health facilities were technically efficient. Besides, the studies conducted in Gambia [53] revealed that only 22% of health centres were technically efficient. This might be due to the use of a lower number of outputs in the earlier study, which might result in a lower proportion of technically efficient health facilities [55]. This study quantified the amount of input to be saved to make inefficient hospitals efficient. Accordingly, inefficient hospitals could save US$ 7271.76 from non-salary recurrent costs and US$ 1308.81 from salary costs per year if they were operating efficiently. This could cover the health expenditure for 260 clients according to the Ethiopian per capita health expenditure, US$ 33, as indicated in the seventh Ethiopian health account [56], and for 215 clients according to the WHO report on per capita health expenditure for low-income countries, US$ 40 [57].

This study also illustrated that all the scale inefficient hospitals are performing below their size. Hence, they have to increase their size of operation to be efficient. The sources of inefficiency would be from both the demand side and the supply side. From the demand side, this might be due to the bypassing of the patients at the primary hospitals to seek care in the general and specialized hospitals [58,59]. And from the supplier side, the high resource allocated to Ethiopian primary health care is not matched to their performance that they are performing below the expected level. That might be a source of inefficiency and the hospitals should increase their capacity of operations to be scale efficient.

The study showed that a 100,000 increase in the catchment population of the hospital increases the technical efficiency score by 0.0341. This finding is similar to the study conducted in Southwest Ethiopia [17] where a unit increase in the catchment population increases the technical efficiency score of health centres and health posts by 0.00002 and 0.0006, respectively. This might be due to the increase in health service users of a health facility as its catchment population increases and so do the service outputs. The presence of incentive packages for the clinical staff increases the technical efficiency score of hospitals by 0.19. This might be due to the positive reinforcing effect of incentives on the productivity of the employees.

The presence of a health facility that provides neonatal health services near the hospital decreases the technical efficiency score by 0.21, which is contrary to the study in Northwest Ethiopia [16], where the presence of a health facility within less than 2km radius increases the technical efficiency score of hospitals by 0.697. This could be due to the decrease in health service users in one of the two hospitals when the distance between the two health facilities decreases; this might decrease the service outputs, which results in lower efficiency scores for hospitals in the current study. The technical efficiency score of the hospitals decreases by 0.14 when the distance from the manager’s residence to the hospital is less than 2km. This might be due to the decrease in the working time of the manager when his/ her home is near to the office.

The technical efficiency scores of hospitals increase by 0.16 when the educational status of the manager is master and above. This finding is supported by the study in Northwest Ethiopia [16] where the educational status of the manager with masters and above increases the technical efficiency of public hospitals by 0.219. This might be due to the efficient use of health service resources by the managers who are more educated and have enough knowledge and skills, to use scarce resources in different settings.

Although this study has many roles in health policy decisions, it has its limitations. First, the study used the input and output data of neonatal health services for one year only; therefore, the efficiency scores may be changed when the data are changed. Second, since the DEA was not adjusted for the hospital’s case mix and quality of service, the efficiency scores in this study might be overestimated. In addition, a neonatal referral was used as a measure of neonatal health services output which might not directly reflect the performance of the hospitals. Finally, the number of DMUs included in this study was not adequate though a trial was made to include a higher proportion DMUs from the source population, 15 primary hospitals out of 18 (83%) were included, than the earlier similar studies in Northwest Ethiopia [16] and Southwest Ethiopia [17] that included 15% and 60% of the source population, respectively. Despite these limitations, this study can serve to evaluate the performance of the health facilities and it can be used as a baseline for further studies in this regard.

## Conclusion

The result of this study showed that most of the studied primary hospitals in northwest Ethiopia were technically efficient. However, more than half of the primary hospitals were scale inefficient. Technically inefficient hospitals wasted a considerable amount of non-salary recurrent costs, salary costs, and beds for neonatal health services. Besides the inefficient hospitals are performing below their size indicating that they have to increase their scale of operation to be efficient. This can be done by increasing the clients’ flow to primary hospitals and improving the performance of the health care workers up to their maximum capacity.

The technical efficiency of neonatal health services provision in the primary hospitals was found to be higher if the hospital had a higher catchment population, a manager with a master’s degree and above, and incentive packages for the clinical staff. On the other hand, technical efficiency was lower when the distance from the manager’s residence to the hospital was less than 2km and the presence of another health facility that provides neonatal health services near the hospital. Redistributing the under-loaded health professionals from the inefficient health facilities to others has to be considered than minimizing the monthly salary of the under-paid health professionals. Moreover, the distance between two or more hospitals and the catchment population they will cover has to be considered to use their maximum potential and allocate health service resources productively. It is better to incorporate a higher number of DMUs, use more than one year of data of health facilities, and add a qualitative dimension in the data to investigate and generate strong evidence on the technical efficiency of neonatal health services provision and other similar health services interventions.

## Supporting information

S1 File (XLSX)

S2 File (DTA)
